# Toxin production in a rare and genetically remote cluster of strains of the *Bacillus cereus *group

**DOI:** 10.1186/1471-2180-7-43

**Published:** 2007-05-21

**Authors:** Annette Fagerlund, Julien Brillard, Rainer Fürst, Marie-Hélène Guinebretière, Per Einar Granum

**Affiliations:** 1 Norwegian School of Veterinary Science, Department of Food Safety and Infection Biology, PO Box 8146 Dep, N-0033 Oslo, Norway; 2 Institut National de la Recherche Agronomique (INRA), UMR 408, Sécurité et Qualité des Produits d'Origine Végétale, Avignon, F-84914, France; Univ Avignon, Avignon, F-84029, France

## Abstract

**Background:**

Three enterotoxins are implicated in diarrhoeal food poisoning due to *Bacillus cereus*: Haemolysin BL (Hbl), Non-haemolytic enterotoxin (Nhe), and Cytotoxin K (CytK). Toxin gene profiling and assays for detection of toxin-producing stains have been used in attempts to evaluate the enterotoxic potential of *B. cereus *group strains. *B. cereus *strain NVH 391/98, isolated from a case of fatal enteritis, was genetically remote from other *B. cereus *group strains. This strain lacked the genes encoding Hbl and Nhe, but contains CytK-1. The high virulence of this strain is thought to be due to the greater cytotoxic activity of CytK-1 compared to CytK-2, and to a high level of *cytK *expression. To date, only three strains containing *cytK-1 *have been identified; *B. cereus *strains NVH 391/98, NVH 883/00, and INRA AF2.

**Results:**

A novel gene variant encoding Nhe was identified in these three strains, which had an average of 80% identity in protein sequence with previously identified Nhe toxins. While culture supernatants containing CytK and Nhe from NVH 391/98 and INRA AF2 were highly cytotoxic, NVH 883/00 expressed little or no CytK and Nhe and was non-cytotoxic. Comparative sequence and expression studies indicated that neither the PlcR/PapR quorum sensing system, nor theYvrGH and YvfTU two-component systems, were responsible for the observed difference in toxin production. Additionally, phylogenetic analysis of 13 genes showed that NVH 391/98, NVH 883/00, and INRA AF2 comprise a novel cluster of strains genetically distant from other *B. cereus *group strains.

**Conclusion:**

Due to its divergent sequence, the novel *nhe *operon had previously not been detected in NVH 391/98 using PCR and several monoclonal antibodies. Thus, toxigenic profiling based on the original *nhe *sequence will fail to detect the toxin in this group of strains. The observation that strain NVH 883/00 carries *cytK-1 *but is non-cytotoxic indicates that the detection of this gene variant is not a sufficient criterion for identification of highly cytotoxic strains. The presence of the novel *nhe *operon and the *cytK-1 *gene variant in this cluster of strains reflect their phylogenetically remote relationship towards other *B. cereus *group strains.

## Background

*Bacillus cereus *is a common cause of bacterial foodborne disease, characterized by either emetic or diarrhoeal syndromes [1]. Three chromosomally encoded toxins are generally linked to diarrhoeal illness: Haemolysin BL (Hbl) [2], Non-haemolytic enterotoxin (Nhe) [3] and Cytotoxin K (CytK) [4]. Hbl and Nhe are three-component toxins composed of proteins L_2_, L_1 _and B, and NheA, NheB and NheC, respectively. The genes encoding all three enterotoxins are found to a similar extent in most species of the *B. cereus *group [5,6], and their expression is positively regulated by the PlcR/PapR quorum sensing system [7,8].

*B. cereus *NVH 391/98, isolated in 1998 from an outbreak causing fatal enteritis, has been shown to express neither Hbl nor Nhe [9], and was the strain in which CytK was first identified [4]. Phylogenetic studies have shown that this strain is placed uniquely distant from main *B. cereus *group clusters [10]. It is currently being subjected to complete genome sequencing by the DOE Joint Genome Institute (USA). We previously found that this strain carried a particularly cytotoxic variant of the CytK protein, named CytK-1, which partly explained why NVH 391/98 was highly pathogenic [11]. Results also indicate that the high cytotoxicity of this strain is a result of an exceptionally high level of *cytK *expression [12]. In earlier studies, in all other strains identified to carry *cytK*, the toxin existed as a different, less cytotoxic variant, named CytK-2. Recently, we identified two additional *B. cereus *strains carrying *cytK-1*: NVH 883/00 and INRA AF2 [13]. In a study performed to elucidate the genetic structure of the *B. cereus *group, these three strains appear to constitute a cluster genetically remote from all other tested strains (M-H. Guinebretière and C. Nguyen-The, unpublished results). While NVH 391/98 and INRA INRA AF2 were highly cytotoxic, NVH 883/00 was in initial experiments shown to be non-toxic towards Vero cells. The aim of this study was to investigate and compare the strains of this rare genetic group, to potentially gain insight into mechanisms responsible for the dramatic differences in cytotoxicity between strains.

## Results

### Strains carrying *cytK-1 *have varying levels of toxicity towards Vero cells

**Figure 1:**
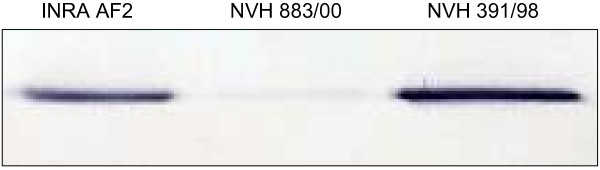
**Detection of CytK in culture supernatants**. Western immunoblot probed with antiserum against CytK. The samples are supernatants from *B. cereus *strains INRA AF2, NVH 883/00 and NVH 391/98, collected from late log phase cultures grown at 32°C.

Supernatants collected from late log phase cultures of strains NVH 391/98 and INRA AF2 grown at 32°C and 37°C, as well as strain NVH 391/98 grown anaerobically at 32°C, gave 100% inhibition of protein synthesis in the Vero cell assay, showing that these strains were highly cytotoxic. In contrast, the supernatants tested from NVH 883/00, obtained from cultures grown at 37°C, 32°C, and 25°C, the latter concentrated by a factor of 100, as well as cultures grown anaerobically at 32°C, were shown to have undetectable toxicity in this assay. To determine whether differential CytK expression could be responsible for the varying levels of toxicity, Western immunoblotting of supernatants from all three strains grown at 32°C were performed using CytK antiserum. The results presented in Figure 1 show that NVH 883/00 expressed substantially less CytK than did NVH 391/98 and INRA AF2.

### A novel variant of Nhe was identified in all three strains

**Figure 2:**
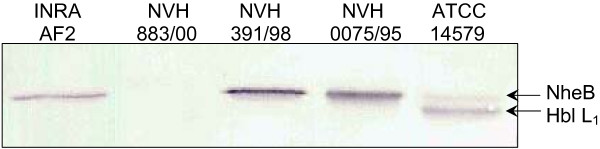
**Detection of NheB in culture supernatants**. Western immunoblot of culture supernatants from *B. cereus *strains INRA AF2, NVH 883/00, NVH 391/98, NVH 0075/95 and ATCC 14579 detected using the 1C2 antibody, reactive against both NheB and Hbl component L_1_. Samples were collected from late log phase cultures grown at 32°C. NVH 0075/95 and ATCC 14579 were used as controls; NVH 0075/95 does not contain *hbl*, while ATCC 14579 produces both Nhe and Hbl. The results show that strains NVH 391/98 and INRA AF2 produce NheB.

Both strains NVH 391/98 and NVH 883/00 were negative in the Tecra and the Oxoid assays, which are commercial kits that employ monoclonal antibodies directed against NheA and Hbl component L_2_, respectively, to detect the presence of Nhe and Hbl in *B. cereus *culture supernatants. Furthermore, all three strains were negative in Western immunoblots using monoclonal antibodies directed against NheA and Hbl component B. However, strains NVH 391/98 and INRA AF2, but not NVH 883/00, were positive in a Western immunoblot using an antibody that is reactive towards both NheB and Hbl component L_1_, giving a band of the same size as NheB (Figure 2). This was quite unexpected as strain NVH 391/98 had previously been shown to not contain the genes encoding Nhe [4,9]. PCR experiments using several sets of primers designed on the basis of published sequences of *nhe *were positive for all three strains with a few of the primer pairs used, thus providing us with a starting point for obtaining the complete sequences of *nhe *in these strains (see Additional file 1). DNA sequencing of the *nhe *operons in strains NVH 391/98 and NVH 883/00 revealed a novel variant of *nhe*, 99.6% identical in DNA sequence between the two strains. The deduced sequences of the three proteins comprising the Nhe toxin, NheA, NheB and NheC, from NVH 391/98 and NVH 883/00 were 77–81%, 87–88%, and 72–74% identical, respectively, to the corresponding protein sequences in *B. cereus *E33L, ATCC 14579, ATCC 10987, G9241, *Bacillus thuringiensis *serovar konkukian str. 97–27, serovar israelensis ATCC 35646, and *Bacillus weihenstephanensis *KBAB4. In comparison, the identities between the Nhe proteins in this set of strains were 96–99%, 97–100%, and 91–99%, respectively.

### The PlcR transcriptional regulator cannot account for varying levels of toxin expression

The PlcR/PapR quorum sensing system is presently the only established regulator of extracellular virulence factors in *B. cereus *group strains [7,8]. Transcription of *cytK *from strain NVH 391/98 has previously been shown to be PlcR-dependent, even though the *cytK *promoter in this strain contains a PlcR recognition sequence with one base divergence from the currently defined PlcR recognition sequence [12]. However, as the *cytK *promoter regions of strains NVH 391/98 and NVH 883/00 are 100% identical, differences in the promoter regions cannot account for the differential expression of CytK in these two strains. Also, a perfect PlcR recognition sequence was found upstream of *nheA *in both strains NVH 391/98 and NVH 883/00.

To explore whether differences in the PlcR/PapR system itself could be responsible for the observed differential expression of CytK and Nhe, *plcR *and *papR *were sequenced in strains NVH 391/98, NVH 883/00, and INRA AF2. The deduced amino acid sequences of both PlcR and PapR were 100% identical in the three strains. Furthermore, the expression of PlcR was determined to be of comparable level in all three investigated strains using Western immunoblotting (results not shown).

### YvrGH and YvfTU two-component systems are not responsible for the varying toxin levels

Regulation of virulence gene expression by two-component systems has been described in a wide range of pathogenic bacteria [14]. A partial sequence encoding a protein with homology towards the YvrG histidine kinase in ATCC 14579 was previously identified to be located upstream of *cytK *in NVH 391/98 [4], while in ATCC 14579, genes BC_5352 and BC_5353 encoding a putative two-component system (*yvfTU*) are located upstream from *plcR *[15]. As two-component systems sometimes control the expression of genes located in the same locus, we explored whether these two-component systems could have a regulatory function explaining the observed differential expression of toxins. *yvrGH *and *yvfTU *were DNA sequenced in both strains NVH 391/98 and NVH 883/00.

The deduced amino acid sequences of YvrH and YvrG each differed with only one amino acid between strains NVH 391/98 and NVH 883/00. A knockout mutant was obtained in *B. cereus *ATCC 14579. Unfortunately, the mutant could not be prepared in NVH 391/98, as all attempts to transform this strain failed despite various conditions used [12]. However, no difference between the *B. cereus *ATCC 14579 wild-type and the *yvrGH *null mutant could be observed in Western immunoblots where culture supernatants were probed using CytK antiserum, in the Vero cell cytotoxicity assay, or in transcriptional assays of *cytK *expression using *lacZ *fusions (results not shown).

**Figure 3:**
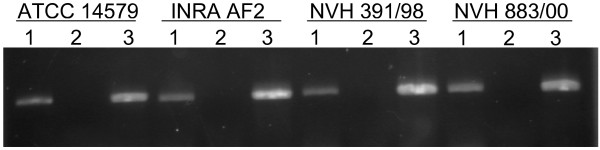
**Detection of *yvfTU *expression by RT-PCR**. Reverse transcriptase (RT) PCR experiments performed on the *yvfTU *operon of *B. cereus *strains ATCC 14579, INRA AF2, NVH 391/98, and NVH 883/00. The operon encodes the YvfTU two-component system. Lanes 1: RT-PCR on 500 ng RNA. Lanes 2: Negative control (RT-PCR on 500 ng RNA with a heat-inactivated reverse-transcriptase). Lanes 3: Positive control (PCR on 200 ng genomic DNA). The ~700 bp PCR product covered the 3'-region of *yvfT *running into the 5'region of *yvfU*, indicating that these two genes were expressed in a single operon.

Unpublished results (by J. Brillard) indicate that a knockout mutant of the *yvfTU *two-component system in *B. cereus *ATCC 14579 showed reduced expression of CytK using *lacZ *fusions, compared with the wild-type strain. However, the *yvfTU *locus located immediately upstream from *plcR *was 100% identical in strains NVH 391/98 and NVH 883/00. Reverse transcription (RT) PCR experiments were carried out to assess whether or not YvfTU was expressed in the low toxin-producing strain NVH 883/00. The results in Figure 3 show that *yvfTU *was expressed in all strains. Thus, neither the YvrGH nor the YvfTU two-component systems seemed to be responsible for the divergent expression of toxins in the presently investigated *B. cereus *strains.

### The three investigated strains are genetically remote from other strains of the *B. cereus *group

**Figure 4:**
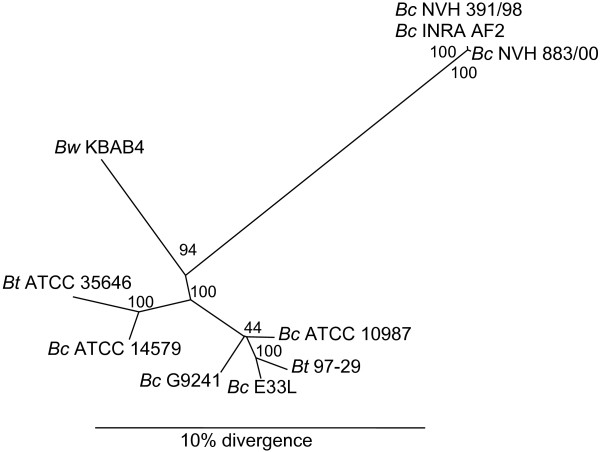
**Phylogenetic analysis**. Neighbour-joining phylogenetic tree prepared from the concatenated housekeeping gene sequences of different strains of the *B. cereus *group. The tree was based on the MLST scheme described at the University of Oslo's *Bacillus cereus *group MultiLocus Sequence Typing website [16]. Bootstrap support values (in %) are shown next to the appropriate nodes. Abbreviations: *Bc*: *B. cereus*, *Bt*: *B. thuringiensis*, *Bw*: *B. weihenstephanensis*.

During the course of this study, 13 genes were DNA sequenced in strains NVH 391/98 and NVH 883/00. It became apparent that while all examined genes were of high identity (> 99%) in NVH 391/98 and NVH 883/00, their identity towards other *B. cereus *strains from which DNA sequences are publicly available was typically around 70–80%. Using PCR experiments, INRA AF2 was confirmed to contain the same gene variants as NVH 391/98 and NVH 883/00. A total of 47 PCR reactions were performed, covering all 13 investigated genes, using a combination of 62 primers specific to strains NVH 391/98 and NVH 883/00 (see Additional file 1). To further study the genetic relatedness of these three strains towards other strains of the *B. cereus *group, multilocus sequence typing (MLST) was performed, using partial sequences of the seven housekeeping genes *adk*, *ccpA*, *glpF*, *glpT*, *panC*, *pta*, and *pycA*. The MLST scheme used is from the University of Oslo's *Bacillus cereus *group MultiLocus Sequence Typing website [16], which is adapted from three previously published schemes [10,17,18]. The obtained sequences from strains NVH 391/98 and INRA AF2 were 100% identical, while the only difference between the sequences of these two strains and NVH 883/00 were one nucleotide change in each of *panC *and *pta*. Figure 4 shows the phylogenetic tree prepared from the sequences obtained from the three examined strains and the corresponding sequences from other *B. cereus *group strains. The results clearly show that strains NVH 391/98, NVH 883/00, and INRA AF2 comprise a genetically remote cluster of strains within the *B. cereus *group.

## Discussion

In the present study, we have investigated aspects of cytotoxicity, toxin production and regulation in a genetically remote and rare collection of strains within the *B. cereus *group. The original strain of this type, *B. cereus *NVH 391/98, was highly pathogenic and the strain in which CytK was first discovered [4], while the closely related *B. cereus *NVH 883/00 and INRA AF2 are the only two other identified strains of this group.

We have identified a novel variant of the genes encoding Nhe in all three investigated strains. Nhe was previously undetected in NVH 391/98 [4,9], most likely due to the high sequence divergence of the novel Nhe-variant as compared with previously identified Nhe proteins. This would result in lack of detection by PCR and by assays employing monoclonal antibodies lacking cross-reactivity towards epitopes on the novel Nhe variant. This is supported by the discovery by Dietrich *et al*. [19] that when monoclonal antibodies were raised against NheB purified from *B. cereus *B-4ac, 20 out of 25 of the obtained antibodies showed reactivity towards an exoprotein from NVH 391/98 of the same size as NheB.

While strains NVH 391/98 and INRA AF2 were shown to be highly cytotoxic towards Vero cells and expressed high levels of the toxins CytK and NheB, strain NVH 883/00 was shown to be non-toxic under all conditions tested, with low or absent toxin expression. To date, these are the only three strains that have been identified to carry the *cytK-1 *variant [13]. The presence of CytK-1 was implicated as the primary reason for the high pathogenicity of NVH 391/98, due to the greater cytotoxic activity of CytK-1 compared with the CytK-2 variant [11], and a particularly high level of *cytK-1 *expression [12]. Although these factors certainly still seem valid, the identification of the non-toxic NVH 883/00 carrying *cytK-1 *indicates that the presence of this gene variant is not a sufficient criterion for identification of highly cytotoxic strains.

It has been proposed that gene content plays a small role in the diversity of phenotype and pathogenicity observed between *B. cereus *group strains, and that subtle changes to regulatory networks are of much greater importance [20]. Selected regulatory genes were investigated and compared, to potentially identify mechanisms responsible for the differential expression of toxins in NVH 391/98 and NVH 883/00. The PlcR/PapR quorum sensing system is presently the only established regulator of expression of extracellular virulence factors in *B. cereus *group strains [7,8]. However, as the deduced PlcR and PapR proteins were 100% identical between strains NVH 391/98 and NVH 883/00, and the expression of PlcR was determined to be of comparable level, we propose that additional regulatory factors are involved.

Two-component systems have often been shown to play key roles in regulation of virulence in bacteria [14,21]. In the current study, the possibility that two selected two-component systems, YvrGH and YvfTU, had a regulatory function related to toxin production was explored. Both were initially chosen for investigation based on their proximity to *cytK-1 *and *plcR*, respectively, as genes under the control of a two-component system often map in the vicinity of the regulatory genes [22]. However, neither the YvrGH nor the YvfTU two-component systems seemed to be responsible for the divergent expression of toxins in the presently investigated *B. cereus *strains, even though YvfTU has been implicated in positive regulation of CytK expression in *B. cereus *ATCC 14579 (J. Brillard, unpublished results).

Phylogenetic analysis (MLST) of strains NVH 391/98, NVH 883/00, and INRA AF2 compared to other *B. cereus *group strains showed that these three strains comprise a genetically remote cluster of strains within the *B. cereus *group. Thus, the presence of the novel *nhe *operon and the *cytK-1 *gene variant in these strains are intrinsic to the distant phylogenetic relationship towards other strains of the *B. cereus *group. Previous phylogenetic studies by Sorokin *et al*. [10] places strain NVH 391/98 rather far from main *B. cereus *group strain clusters. Based on these data, one could propose that the presently studied *B. cereus *strains should comprise a novel species. The assignment of a new species has probably been hampered by the fact that prior to the current report, only one single strain of this cluster (NVH 391/98) has been described, and perhaps that these strains do not seem to be distinguishable from other *B. cereus *strains based on traditional criteria such as morphology and phenotype.

## Conclusion

We have discovered a novel Nhe variant in a rare group of *B. cereus *strains, which has previously been undetected in culture supernatants using PCR and methods based on monoclonal antibodies, due to its divergent sequence as compared with previously identified Nhe proteins. Despite *cytK-1 *expression having been shown to be exceptionally high in strain NVH 391/98 [12], the presence of a *cytK-1 *gene alone does not imply that the organism has high pathogenic potential, since strain NVH 883/00 carries *cytK-1 *but is non-cytotoxic. However, the presence of the novel *nhe *operon and the *cytK-1 *gene variant in strains NVH 391/98, NVH 883/00 and INRA AF2 seems to be a characteristic that is linked to their distant genetic relationship towards other *B. cereus *group strains. Furthermore, we found no evidence that the PlcR/PapR system or the YvrGH and YvfTU two-component systems are responsible for the differing levels of Nhe and CytK toxin expression between these strains. We therefore postulate that there must be additional unidentified regulatory mechanisms that are of significant importance for regulation of toxin expression in *B. cereus*.

## Methods

### Strains and growth conditions

*B. cereus *NVH 391/98 is a clinical isolate [4]. *B. cereus *NVH 883/00 is an isolate from spices and *B. cereus *NVH 0075/95 is a food-poisoning strain, both submitted to the Norwegian School of Veterinary Science, Oslo, Norway. *B. cereus *INRA AF2 is an isolate connected to a food poisoning case, submitted to the Institut National de la Recherche Agronomique, Avignon, France. *B. cereus *ATCC 14579 was used for construction of the *yvrGH *knockout mutant. XL10-Gold *E. coli *(Stratagene) was used for recombinant expression of CytK-1. *B. cereus *was grown in BHIG (brain heart infusion supplemented with 1 % w/v glucose) at 32°C or 37°C and 200 rpm, from overnight cultures diluted 1:100. Supernatants were collected by centrifugation. Supernatant from cultures grown at 25°C was concentrated 100 times by precipitation with 70% saturated (NH_4_)_2_SO_4_. Anaerobic growth was performed as follows: 5 ml of BHIG was boiled in glass tubes to remove oxygen, cooled, and inoculated with 50 μl of an overnight culture. The tubes were incubated at 32°C under anaerobic conditions, and samples of supernatants were obtained after 6, 24 and 48 hours.

### Cytotoxicity assays

Cytotoxicity was determined using a Vero cell test [23]. The assay monitors the inhibition of protein synthesis in the cells by measuring the reduction of incorporated ^14^C-leucine in the Vero cells upon addition of toxin proteins. 100 μl samples of late log phase culture supernatants were applied to the assay in duplicate. The *B. cereus *Enterotoxin Reverse Passive Latex Agglutination test kit (Oxoid, Bassingstoke, England) and the *Bacillus *Diarrheal Enterotoxin visual immunoassay (Tecra Diagnostics, Roseville, Australia) were used according to the manufacturers' instructions.

### PCR and DNA sequencing of toxin and regulatory genes

Genomic DNA from *B. cereus *used as template in PCRs was isolated using the method of Pospiech and Neumann [24]. PCR products subjected to DNA sequencing were obtained either by conventional PCR, inverse PCR, or using the DNA Walking SpeedUp premix kit (Seegene). DNA sequencing was performed on both strands and sequences were submitted to GenBank, with the following accessions for the NVH 883/00 sequences: [GenBank:DQ885233] for the locus containing *yvrG*, *yvrH*, the *orf2 *upstream of *cytK-1 *and *cytK-1*, [GenBank:DQ885234] for the locus containing the gene encoding an ABC transporter permease protein, *yvfT*, *yvfU*, *plcR*, and *papR*, and [GenBank:DQ885235] for *nheA*, *nheB*, *nheC*, and the downstream gene encoding a deoxyribonucleotide regulator. The corresponding DNA sequences obtained from strain NVH 391/98 in this study were identical to the corresponding sequences of the NVH 391/98 shotgun genome sequence [GenBank:AALL00000000], except in the case of the *nhe *operon, in which the complete sequence was obtained in the current report [GenBank:DQ885236]. The *plcR *and *papR *genes from strain INRA AF2 has the following accession: [GenBank:EF108376].

### Sequence and phylogenetic analysis

The positions of coding sequences were predicted using EasyGene [25]. The following genomic sequences were used as reference strains: *B. cereus *ATCC 14579 [GenBank:AE016877], *B. cereus *ATCC 10987 [GenBank:AE017194], *B. cereus *G9241 [GenBank:AAEK01000000], *B. cereus *E33L [GenBank:CP000001], *B. thuringiensis *serovar konkukian str. 97–27 [GenBank:AE017355], *B. thuringiensis *serovar israelensis ATCC 35646 [GenBank:AAJM01000000], and *B. weihenstephanensis *KBAB4 [GenBank:AAOY00000000]. Pairwise alignments were calculated using the Smith-Waterman algorithm [26].

MLST was performed using partial sequences of the genes *adk*, *ccpA*, *glpF*, *glpT*, *panC*, *pta*, and *pycA*, ranging in length from 330 to 444 nucleotides, according to the scheme given at the University of Oslo's *Bacillus cereus *group MultiLocus Sequence Typing website [16]. PCR and DNA sequencing was performed as described, with the exception that primer aagtaagggctaagaaga was used as the forward primer for amplification of *glpT*, and primers ccaagggatataaagcgagatg and aatcaactataccgtttgtatttgc was used for *panC*. The GenBank accessions for the sequences from NVH 883/00 are *adk*; [GenBank:EF108377], *ccpA*; [GenBank:EF108378], *glpF*; [GenBank:EF108379], *glpT*; [GenBank:EF108380], *panC*; [GenBank:EF108381], *pta*; [GenBank:EF108382], and *pycA*; [GenBank:EF108383]. The GenBank accessions for the sequences from INRA AF2 are *adk*; [GenBank:EF108384], *ccpA*; [GenBank:EF108385], *glpF*; [GenBank:EF108386], *glpT*; [GenBank:EF108387], *panC*; [GenBank:EF108388], *pta*; [GenBank:EF108389], and *pycA*; [GenBank:EF108390]. The sequences for strain NVH 391/98 were obtained from the NVH 391/98 shotgun genome sequence [GenBank:AALL00000000], except in case of the *adk *locus, for which the sequence was obtained in the current report [GenBank:EF108391]. The reference strains listed above were used for all loci, with the exception of *glpF*, which was absent from the available shotgun genome sequences of *B. weihenstephaniensis *KBAB4 and *B. thuringiensis *serovar israelensis ATCC 35646, and tuncated in *B. cereus *G9241. The DNA sequences of all seven genes were concatenated, giving a sequence 2700 nucleotides in length, and aligned using ClustalW. A phylogenetic tree was calculated using the neighbour-joining method, where genetic distances were estimated using the Kimura model and bootstrap confidence values were generated using 1000 permutations. The tree was printed using TreeView [27].

### Preparation of CytK antiserum

Recombinant CytK-1 for use in antiserum production was expressed from the vector construct pMS20-*cytK-1 *previously described [11]. Cultures were grown in 2 litres of modified CGY medium [28] containing 0.4 % glucose and 50 μg ml^-1 ^kanamycin, and incubated at 37°C for 5 hours. The harvested pellet was resuspended in 20 ml lysis buffer (50 mM NaH_2_PO_4_, 300 mM NaCl, pH 8.0, 1 mg ml^-1 ^lysozyme). Incubated on ice 30 minutes, and lysed by sonication. 0.25 mg RNase A and 10 U DNase (Promega) was added, followed by incubation on ice for 20 minutes. Purification of CytK-1 protein from the supernatant was performed as previously described [4]. A N2W rabbit was immunized using four ~1 ml doses, administered in two-week intervals, with a final bleeding six days after the fourth immunization. Fresh protein samples were used for each immunization.

### SDS-PAGE and Western immunoblotting

SDS-PAGE (12% acrylamide) and Western immunoblotting was carried out according to standard protocols [29]. For detection of enterotoxins by Western immunoblotting, 12 μl culture supernatant from late log phase cultures grown at 32°C was applied. CytK antiserum was used at a dilution of 1:500. For detection of PlcR, bacterial pellet from 100 μl culture was suspended in SDS-PAGE sample buffer and applied. The PlcR rabbit antiserum [30], diluted 1:2000, was kindly supplied by Dr. Stephen Leppla, Maryland, USA. The secondary antibody biotin-goat-anti-rabbit IgG (Bio-Rad) was used at a 1:1000 dilution. Monoclonal antibodies 1A8 against NheA [19], 2A3 against Hbl component B, and 1C2 against both NheB and Hbl component L_1 _[31], used at dilutions of 1:15, were obtained as a gift from Dr. Erwin Märtlbauer, University of Munich, Germany. For these blots, the secondary antibody biotin-goat-anti-mouse IgG (Amersham Biosciences) was used at a 1:500 dilution. All blots were incubated with a complex of streptavidin and biotinylated alkaline phosphatase used at a dilution of 1:3000, prior to development with a NBT/BCIP solution.

### Construction of the *yvrGH *mutant

The *yvrGH *operon was interrupted by allelic exchange with a kanamycin resistance gene (Km^R^) in *B. cereus *ATCC 14579 as follows: The regions upstream of *yvrH *and downstream of *yvrG *were PCR amplified using tgcaggatccgttagcaaatcgccactact/tgcagtcgacatacgtagttcggattctcg and tgcactgcagagaatggattaccggtctaac/tgcaccatggagggaagcaggttagtattgt, respectively. PCR products were digested with *Bam*HI/*Sal*I and *Pst*I/*Nco*I using primer-incorporated restriction sites (underlined). Km^R ^was PCR-amplified from pDG783 [32] using primers tctggtcgaccatttgaggtgatagg and gctactgcagatcgatacaaattcctcgtaggcg, and digested with *Sal*I/*Pst*I. The three digested DNA fragments were purified, and ligated into *Nco*I/*Bam*HI digested pMAD [33]. The recombinant plasmid pMADΔ*yvrGH *was transformed into *B. cereus *ATCC 14579, transformants were subjected to allelic exchange as previously described [33], and the *yvrGH *mutant was confirmed by DNA sequencing.

### *cytK *transcriptional activity

The *cytK*-promoter of *B. cereus *ATCC 14579 has previously been cloned in pHT304-18'Z, giving a *cytK'-lacZ *transcriptional fusion [12]. The plasmid was transformed into *B. cereus *ATCC 14579 wild-type and Δ*yvrGH*, and cells were grown in LB medium at 37°C with shaking. β-galactosidase specific activities were measured in triplicate samples from each culture as described previously [34]. Experiments were repeated twice.

### RT-PCR of *yvfTU*

RNA extraction was performed using the FastRNA ProBlue kit (Qbiogen) on strains grown at 37°C in LB medium and harvested in stationary phase (OD_600 _= 1.4). RT-PCR experiments were performed with the Titan One Tube RT-PCR System (Roche), using 500 ng RNA in each reaction. The primers used were either ttgtGaaAaatccagagcgtgc (for ATCC 14579) or ttgtAaaGaatccagagcgtgc (for the three clinical strains), and atccaatccactttgaatcggc. Diverging nucleotides in the forward primer sequences are indicated in uppercase.

## Authors' contributions

AF drafted the manuscript and participated in the design of the study, did cytotoxicity assays, antiserum preparation and immunoblotting, PCRs and DNA sequencing, and the sequence and phylogenetic analysis. JB constructed the *yvrGH *mutant, did the transcriptional assays, the RT-PCR experiments, and contributed to writing. RF contributed to the cytotoxicity assays, the PCRs and DNA sequencing, and the immunoblotting. MHG provided the INRA AF2 strain and critically revised the manuscript. PEG conceived of the study, participated in its design and critically revised the manuscript. All authors read and approved the final manuscript.

## Supplementary Material

Additional file 1 Supplimentary data Click here for file

## References

[B1] Granum PE, Lund T (1997). *Bacillus cereus *and its food poisoning toxins. FEMS Microbiol Lett.

[B2] Beecher DJ, MacMillan JD (1991). Characterization of the components of hemolysin BL from *Bacillus cereus*. Infect Immun.

[B3] Granum PE, O'Sullivan K, Lund T (1999). The sequence of the non-haemolytic enterotoxin operon from *Bacillus cereus*. FEMS Microbiol Lett.

[B4] Lund T, De Buyser ML, Granum PE (2000). A new cytotoxin from *Bacillus cereus *that may cause necrotic enteritis. Mol Microbiol.

[B5] Mendelson I, Tobery S, Scorpio A, Bozue J, Shafferman A, Friedlander AM (2004). The NheA component of the non-hemolytic enterotoxin of *Bacillus cereus *is produced by *Bacillus anthracis *but is not required for virulence. Microb Pathog.

[B6] Stenfors LP, Mayr R, Scherer S, Granum PE (2002). Pathogenic potential of fifty *Bacillus weihenstephanensis *strains. FEMS Microbiol Lett.

[B7] Gohar M, Økstad OA, Gilois N, Sanchis V, Kolstø AB, Lereclus D (2002). Two-dimensional electrophoresis analysis of the extracellular proteome of *Bacillus cereus *reveals the importance of the PlcR regulon. Proteomics.

[B8] Slamti L, Lereclus D (2002). A cell-cell signaling peptide activates the PlcR virulence regulon in bacteria of the *Bacillus cereus *group. EMBO J.

[B9] Guinebretière MH, Broussolle V, Nguyen-The C (2002). Enterotoxigenic profiles of food-poisoning and food-borne *Bacillus cereus *strains. J Clin Microbiol.

[B10] Sorokin A, Candelon B, Guilloux K, Galleron N, Wackerow-Kouzova N, Ehrlich SD, Bourguet D, Sanchis V (2006). Multiple-locus sequence typing analysis of *Bacillus cereus *and *Bacillus thuringiensis *reveals separate clustering and a distinct population structure of psychrotrophic strains. Appl Environ Microbiol.

[B11] Fagerlund A, Ween O, Lund T, Hardy SP, Granum PE (2004). Genetic and functional analysis of the *cytK *family of genes in *Bacillus cereus*. Microbiology.

[B12] Brillard J, Lereclus D (2004). Comparison of cytotoxin *cytK *promoters from *Bacillus cereus *strain ATCC 14579 and from a *B cereus *food-poisoning strain. Microbiology.

[B13] Guinebretière MH, Fagerlund A, Granum PE, Nguyen-The C (2006). Rapid discrimination of *cytK-1 *and *cytK-2 *genes in *Bacillus cereus *strains by a novel duplex PCR system. FEMS Microbiol Lett.

[B14] Stephenson K, Hoch JA (2004). Developing inhibitors to selectively target two-component and phosphorelay signal transduction systems of pathogenic microorganisms. Curr Med Chem.

[B15] Økstad OA, Gominet M, Purnelle B, Rose M, Lereclus D, Kolstø AB (1999). Sequence analysis of three *Bacillus cereus *loci carrying PIcR-regulated genes encoding degradative enzymes and enterotoxin. Microbiology.

[B16] University of Oslo's *Bacillus cereus *group MultiLocus Sequence Typing website. http://mlstoslo.uio.no.

[B17] Helgason E, Tourasse NJ, Meisal R, Caugant DA, Kolstø AB (2004). Multilocus sequence typing scheme for bacteria of the *Bacillus cereus *group. Appl Environ Microbiol.

[B18] Priest FG, Barker M, Baillie LWJ, Holmes EC, Maiden MCJ (2004). Population structure and evolution of the *Bacillus cereus *group. J Bacteriol.

[B19] Dietrich R, Moravek M, Burk C, Granum PE, Märtlbauer E (2005). Production and characterization of antibodies against each of the three subunits of the *Bacillus cereus *nonhemolytic enterotoxin complex. Appl Environ Microbiol.

[B20] Rasko DA, Altherr MR, Han CS, Ravel J (2005). Genomics of the *Bacillus cereus *group of organisms. FEMS Microbiol Rev.

[B21] Hoch JA (2000). Two-component and phosphorelay signal transduction. Curr Opin Microbiol.

[B22] Charles TC, Nester EW (1993). A chromosomally encoded two-component sensory transduction system is required for virulence of *Agrobacterium tumefaciens*. J Bacteriol.

[B23] Lindbäck T, Granum PE, Adley CC (2006). Detection and purification of *Bacillus cereus *enterotoxins. Methods in Biotechnology, Food-Borne Pathogens: Methods and Protocols.

[B24] Pospiech A, Neumann B (1995). A versatile quick-prep of genomic DNA from Gram-positive bacteria. Trends Genet.

[B25] Larsen TS, Krogh A (2003). EasyGene – a prokaryotic gene finder that ranks ORFs by statistical significance. BMC Bioinformatics.

[B26] Smith TF, Waterman MS (1981). Identification of common molecular subsequences. J Mol Biol.

[B27] Page RD (1996). TreeView: an application to display phylogenetic trees on personal computers. Comput Appl Biosci.

[B28] Beecher DJ, Wong AC (1994). Improved purification and characterization of hemolysin BL, a hemolytic dermonecrotic vascular permeability factor from *Bacillus cereus*. Infect Immun.

[B29] Harlow E, Lane D (1988). Antibodies: A Laboratory Manual.

[B30] Pomerantsev AP, Kalnin KV, Osorio M, Leppla SH (2003). Phosphatidylcholine-specific phospholipase C and sphingomyelinase activities in bacteria of the *Bacillus cereus *group. Infect Immun.

[B31] Dietrich R, Fella C, Strich S, Märtlbauer E (1999). Production and characterization of monoclonal antibodies against the hemolysin BL enterotoxin complex produced by *Bacillus cereus*. Appl Environ Microbiol.

[B32] Trieu-Cuot P, Courvalin P (1983). Nucleotide sequence of the *Streptococcus faecalis *plasmid gene encoding the 3'5"-aminoglycoside phosphotransferase type III. Gene.

[B33] Arnaud M, Chastanet A, Débarbouillé M (2004). New vector for efficient allelic replacement in naturally nontransformable, low-GC-content, gram-positive bacteria. Appl Environ Microbiol.

[B34] Bouillaut L, Ramarao N, Buisson C, Gilois N, Gohar M, Lereclus D, Nielsen-Leroux C (2005). FlhA influences *Bacillus thuringiensis *PlcR-regulated gene transcription, protein production, and virulence. Appl Environ Microbiol.

