# Supplementary material for: Toxin production in a rare and genetically remote cluster of strains of the Bacillus cereus group
Source: BMC Microbiol. 2007 May 21;7:43. doi: 10.1186/1471-2180-7-43 (PMC1888693; doi:10.1186/1471-2180-7-43)
Supplement: Additional file 1 — Supplimentary data [file 1471-2180-7-43-S1.pdf]

Additional file 1

PCR reaction conditions

For all PCR reactions, the following reaction mixture was used:

1 µl or 0.5 µl genome prep

2.5 µl forward primer (10 pM)

2.5 µl reverse primer (10 pM)

1 µl dNTP mix (10 mM each)

1 µl DyNAzyme II DNA Polymerase (Finnzymes)

5 µl 10× Reaction buffer

37 µl dH_2O

The reactions were subjected to thermal cycling using the following program: Initial denaturing at 95 °C for 3 minutes, then 30 cycles of 95 °C for 1 minute, 45-55 °C (depending on the primers) for 1 minute and 72 °C for 1 minute, followed by an elongation step of 7 minutes at 72 °C.

Initial PCR detection of *nhe* genes in strains NVH 391/98, NVH 883/00, and INRA AF2

The primer pairs based on earlier published sequences of *nhe* that were initially positive for the three strains NVH 391/98, NVH 883/00, and INRA AF2 were the following three forward primers: cggttcatctgttgcgacagc, gcgaagcaatggttagatgta, and acgttacaaacgttgaagtac, and the two reverse primers: tgaattaattttgcgtatgaatccattgc and gcatatgaatccattgcaa. All three forward primers gave positive PCR results in reactions with both reverse primers.

Details of the PCR experiments that confirm that INRA AF2 has the same gene variants as strains NVH 391/98 and NVH 883/00 for 13 genes:

The PCR reactions listed below were positive and of the correct size for strain INRA AF2 using primers designed from the corresponding sequences in NVH 391/98 and NVH 883/00. The targeted genes were *yvrG*, *yvrH*, *orf2* upstream of *cytK*, *cytK*, the gene encoding an ABC transporter permease protein (*ABC*), *yvfT*, *yvfU*, *plcR*, *papR*, *nheA*, *nheB*, *nheC*, and the gene encoding a deoxyribonucleotide regulator (*DeoR*).

Forward primer	Reverse primer	Targeted gene	Size
yvfF6	yvfR6	*ABC*	1640 bp
yvfF11	yvfR7	*ABC*, *yvfT*	1711 bp
yvfF6	yvfR5	*ABC*, *yvfT*	1814 bp
yvfF11	yvfR5	*ABC*, *yvfT*	972 bp
yvfF11	yvfR4	*ABC*, *yvfT*	1199 bp
yvfF11	yvfR3	*ABC*, *yvfT*	1341 bp
yvfF4	yvfR8	*yvfT, yvfU*	1677 bp
yvfF4	yvfR2	*yvfT*, *yvfU*	1388 bp
yvfF2	yvfR7	*yvfT*	933 bp
yvfF13	plcR391R2	*yvfU*, *plcR*	806 bp
plcR391F4	plcR391R2	*plcR* promoter	537 bp
plcR391F2	plcR391R4	*plcR*, *papR*	1367 bp
plcR391F4	plcR391R4	*plcR*, *papR*	1840 bp
plcR391F1	plcR391R4	*plcR*, *papR*	1321 bp
RRf3	RegulatorR2	*yvrH*	671 bp
RRf3	HKr6	*yvrH*, *yvrG*	1743 bp
RRf6	HKr6	*yvrH*, *yvrG*	1118 bp
RRf5	HKr6	*yvrH*, *yvrG*	957 bp
RRf3	HKr6	*yvrH*, *yvrG*	1743 bp
HKf6	HKR1	*yvrG*	895 bp
HKf6	R8	*yvrG*	1451 bp
HK1	R6	*yvrG*	660 bp
HK2	R5	*yvrG*, *orf2*	645 bp
HK1	orf2R4	*yvrG*, *orf2*	1256 bp
HKf6	R4FR	*yvrG*, *orf2*	2009 bp
orf2F1	R3fr	*orf2*, *cytK*	948 bp
orf2F3	Rent3	*orf2*, *cytK*	1734 bp
F3fr	Rent1	*cytK*	627 bp
F6FR	R2cytK	*cytK*	674 bp
Fent1	Rent2	*cytK*	651 bp
Fent1	Rent3	*cytK*	748 bp
Fent2	Rent3	*cytK*	554 bp
nhe391F12	nhe391R13	*nheA* promoter	832 bp
nhe391F12	nhe391R9	*nheA*	1152 bp
nhe391F12	nhe391R8	*nheA*	1864 bp
nhe391F11	nhe391R9	*nheA*	538 bp
nhe391F11	nhe391R8	*nheA*	1246 bp
nhe391F12	nhe391R6	*nheA*, *nheB*	2596 bp
nhe391F11	nhe391R6	*nheA*, *nheB*	1978 bp
nhe391F7	nhe391R7	*nheA*, *nheB*	814 bp
nhe391F9	nhe391R7	*nheA*, *nheB*	1626 bp
nhe391F7	nhe391R4	*nheA*, *nheB*	1050 bp
nhe391F8	nhe391R2	*nheB*	861 bp
nhe391F1	nhe391R14	*nheB*, *nheC*	1267 bp
nhe391F8	nhe391R5	*nheB*, *nheC*	1487 bp
nhe391F2	nhe391R10	*nheC*, *deoR*	1530 bp
nhe391F10	nhe391R11	*deoR*	863 bp

Primer sequences:

F3fr:	aacagatatcggtcaaaatgc
F6FR:	gcattatctgctgtgttggc
Fent1:	agtaacttggtctgattccg
Fent2:	atgaaacagatgcacgaggc
HK1:	gaacaggacttggtctcgc
HK2:	caattgaaagtgaacttggg
HKf6:	gatgaggatgtaatcgcgaaa
HKR1:	cgagaccaagtcctgttcc
HKr6:	tgagccaacgcattgtatgt
nhe391F1:	tgcacaggcagaaattcaaa
nhe391F10:	tgaaatcgctcgaaaaatgg
nhe391F11:	cgttaataaggcgctgtgttt
nhe391F12:	tcagcttcatggtggaattg
nhe391F2:	gcaatggtgcaaacaacatc
nhe391F7:	aaacgaagcagaggtaacagaaa
nhe391F8:	aagatgcaatggaacgcact
nhe391F9:	gcttactacgaaggggagca
nhe391R10:	aagcaatcgtctcatttgcat
nhe391R11:	tgcaatgttcatatctttcactcc
nhe391R13:	acattcagcccttgcactct
nhe391R14:	cggcatcaattgtttctgtc
nhe391R2:	ctcccattgtatgccactga
nhe391R4:	gctgtggctacagaggaacc
nhe391R5:	tgttgtattgcgcttgaaaaa
nhe391R6:	tccctttgtcagagttgcttt
nhe391R7:	caaccttgttaatccctttgtca
nhe391R8:	atcaacgagctccgtaaagc
nhe391R9:	actactcatcgcgctcacct
orf2F1:	gcaacgaaaggttctctcca
orf2F3:	gtctgttatgagcttaggtttgg
orf2R4:	cgaaggaaatgaaagaggaga
plcR391F1:	gatgaggggtttaacgcaaa
plcR391F2:	gcgtgcaagcagaaaagctagg
plcR391F4:	ggtgattttaccgcgaacag
plcR391R1:	ttgctcaccatcccattaca
plcR391R2:	tgcgttaaacccctcatcactc
plcR391R4:	ttgctggggacgatattagc
R2cytK:	gaatacataaataattggtttcc
R3fr:	gtgtttttgcattttgaccg
R4FR:	caccagcgatgactaagcc
R5:	tatgtttcctagggaagagc
R6:	gttcactacaatcactttcc
R8:	ggagagaacctttcgttgc
RegulatorR2:	ttcggattctcgcaaaagaagagt
Rent1:	cgtgcatctgtttcatgagg
Rent2:	tattgttcgttgcaccttgc
Rent3:	aatcgtaataggtgttccgg
RRf3:	tcacactcatctcctcccta
RRf5:	aacccaagcaacccacagta
RRf6:	ccgctcagctatttcagctc
yvfF11:	gcagtggaacgtggaagtct
yvfF13:	ggcactgtatctttctcctggt
yvfF2:	aagaaaaagacaggaagcggta
yvfF4:	gcatttgcaccaacaaaaag
yvfF6:	ggggtggagagatggaaaag
yvfR2:	cgcatcaagcttcgttaatactt
yvfR3:	gcgctcttcgcgttttacta
yvfR4:	atgcgattgcatttgctgta
yvfR5:	ttgccggtatgctagaacaa
yvfR6:	ccgcttcctgtctttttctt
yvfR7:	cgcatgatgtcgccttacta
yvfR8:	gaaatgaagtacgccttatacaaaca
